# Dr. Kreysig

**Published:** 1840-01

**Authors:** 


### FRIEDRICH LUDWIG KREYSIG, M.D., FIRST PHYSICIAN TO THE KING OF SAXONY, &c. &c.

F. L. KREYSIG was born on the 7th July, 1770, at Eilenburg, where his father was a practising physician. In his twelfth year he was sent to the public school of Grimma, where he laid the foundation of his scholarship, and where he remained until 1785, when he went to the university of Leipsig, to study medicine. After four years' residence here, having obtained what we would term a travelling fellowship (Das Kregel-Sternbachsche Stipendium), he went to Pavia, in 1792, and had the benefit of the instruction of Peter Frank, Scarpa, Paletta, and Spallanzani. Returned to Leipsig, he took the degrees of Master of Philosophy, and Doctor of Medicine; and, for some time, exercised himself as a private teacher of medicine. In 1796 he was appointed to the chair of

Pathology and Surgery, as substitute of Dr. Leonhardi, which he retained until 1801, when he was received into the faculty of the university as Professor of Anatomy and Botany. Previously to this appointment, however, he had published his important work, in two volumes, 8vo, entitled "Neue Darstellung der Physiologischen und Pathologischen Grundlehren für angehende Aerzte und Pracktiker." Leipsig, 1798-1800. Kreysig's reputation as a man of science and a skilful practitioner grew so rapidly, that in 1803, while still only in his thirty-third year, he was called to Dresden, as body-physician to the elector, afterwards king of Saxony. From this time, his fortunes were intimately united with those of his royal patron; and, during the eventful years from 1806 to 1815, these often carried him beyond the limits of his native country. In the king's journies to Poland, &c., Kreysig was his constant companion, and, on these occasions, his abilities procured him a degree of reputation throughout these northern regions, which never left him. The leisure he enjoyed in the years 1813-15, during the king's compulsory stay at Berlin and Friedrichsfelde, he devoted to the composition of his great work on Diseases of the Heart, which was published at Berlin, in four volumes, 8vo (1814-18), under the title "Die Krankheiten des Herzens systematisch bearbeitet und durch eigene Beobachtungen erläutert." On his return to Dresden, he published, in 1818-19, his "System der praktischen Heilkunde," in two parts; a work which was never completed. He also took upon himself the professorship of Pathology and Therapeutics, and the direction of the Medical Clinic in the Medico-Chirurgical Academy, which he himself had almost created; besides other high official duties relating to the medical institutions of the kingdom. From this period, his private practice became most extensive; his reputation not being confined to Dresden or Saxony, but extending throughout Europe. Patients, affected with chronic diseases, came to him from all countries, and his consultations, by letter, were probably unexampled in extent. And this reputation was most legitimately acquired, through the superior science and skill, and the most cordial attention of the physician. His success as a practitioner was worthy of his renown; and it is much to be regretted that we possess no permanent record of his views and practice at this period of his life, beyond what is contained in his work on mineral waters, first published in English in 1824, and in the following year at Leipsig, under the title "Ueber den Gebrauch der natürlichen und künstlichen Mineralwasser von Carlsbad, Ems, Marienbad, Eger, Pyrmont, und Spa."

In the year 1822, Kreysig suffered a severe illness, and the consequences of this, and the death of the king, in 1827, led him to resign some of his official appointments, and also to contract the sphere of his practice; and henceforward he gave himself more up to the common amusements of life. His occupations were, however, still of a scientific kind; and he carried to a considerable extent the indulgence of his taste for horticulture and exotic botany. He also never lost sight of the improvement of his two principal works, on Diseases of the Heart, and on Practical Medicine, both of which he was desirous of placing on a level with the actual state of science, and of enriching with the treasures of his own practical experience. These objects, however, were far from being attained; he being only able to leave ready for the press the first volume of the first of these two works. In the year 1838, Dr. K. paid a visit to England, in company with Dr. Chaufepié, of Hamburg, with whom he also visited Ireland, and greatly enjoyed the reception he met with from his brethren in this country. Probably this journey was too much for his strength, or the excitement too great for his nervous system. At any rate, he fell into bad health afterwards, and, on the 27th of the following May (1839), he was seized with erysipelas of the face and head, which terminated fatally, on the 6th of June. On the 7th of the same month, he was buried with great pomp, being followed to the grave by all the most distinguished persons, professional and others, in Dresden: and it may be truly said of him, as it has been said only of the best of men, that, as he lived beloved, he died lamented.

